# Empowerment and enablement and their associations with change in health-related quality of life after a supported osteoarthritis self-management programme – a prospective observational study

**DOI:** 10.1186/s40945-023-00172-7

**Published:** 2023-09-22

**Authors:** Karin Sturesdotter Åkesson, Anne Sundén, Kjerstin Stigmar, Frida Eek, Teresa Pawlikowska, Eva Ekvall Hansson

**Affiliations:** 1 Department of Health Sciences, Faculty of Medicine, Lund University, Lund, Sweden; 2 Health Professions Education Centre, RCSI University of Medicine and Health Sciences, Dublin, Ireland

**Keywords:** Physiotherapist, Patient education, Osteoarthritis, Quality of life, Empowerment, Primary health care

## Abstract

**Background:**

Osteoarthritis is a leading cause of disability worldwide. Current treatment supports coping strategies to improve health-related quality of life (HRQoL). The need to predict response to treatment has been raised to personalise care. This study aims to examine change in HRQoL from baseline to three and nine months follow-up after participating in a Supported Osteoarthritis Self-Management Programme (SOASP) and to examine if empowerment and/or enablement were associated with change in HRQoL after a SOASP.

**Methods:**

Patients participating in a SOASP were recruited consecutively between April 2016 and June 2018. The EQ-5D was used to measure HRQoL, the Swedish Rheumatic Disease Empowerment Scale (SWE-RES-23) (score range 1–5) to measure empowerment and the Patient Enablement Instrument (PEI) (score range 0–12) to measure enablement. The instruments were answered before (EQ-5D, SWE-RES-23) and after (EQ-5D, SWE-RES-23, PEI) the SOASP. A patient partner was involved in the research process to enhance the patient perspective. Changes in outcome were examined with paired sample t-test and standardized effect sizes (Cohen´s *d*). Multiple linear regression analysis was performed to assess potential associations.

**Results:**

One hundred forty-three patients participated in baseline measurement. Mean EQ-5D-5 L index score increased significantly from baseline to three months corresponding to a standardised effect size (Cohen´s *d*) of *d* = 0.43, 95% CI [0.24, 0.63] (*n* = 109), and from baseline to nine months *d* = 0.19, 95% CI [0.01, 0.37] (*n* = 119). The average EQ VAS score increased significantly from baseline to three months corresponding to a standardised effect size of *d* = 0.26, 95% CI [0.07, 0.45] (*n* = 109), and from baseline to nine months *d* = 0.18, 95% CI [0.00, 0.36] (*n* = 119). Neither SWE-RES-23 nor PEI at three months follow-up nor the change in the SWE-RES-23 score from baseline to three months follow-up were associated with change in either EQ-5D-5 L index (*p* > 0.05) or the EQ VAS (p > 0.05).

**Conclusions:**

Health-related quality of life increased after participating in a SOASP. Empowerment and enablement as measured with the SWE-RES-23 and the PEI were not associated with change in HRQoL among patients participating in a SOASP.

**Trial registration:**

ClinicalTrials.gov. Identification number: NCT 02974036. First registration 28/11/2016, retrospectively registered.

**Supplementary Information:**

The online version contains supplementary material available at 10.1186/s40945-023-00172-7.



## Background

Osteoarthritis (OA) is one of the most disabling diseases among older adults [1]. According to worldwide estimates, about 9.6% of men and 18.0% of women over the age of 60 years have symptomatic OA [1]. The demands on the health care system will increase since OA prevalence is rising due to population ageing and an increase in risk factors for developing OA such as obesity [2]. International and national guidelines recommend education, exercise and weight loss (if needed) as first line treatment of OA [3–6].

The Swedish national guidelines for OA recommend a structured care approach where early diagnosis based on patient history and clinical examination is emphasised [6]. All OA patients should be offered first line treatment i.e., patient education, exercise, coping strategies and weight loss if needed [6]. First line treatment for OA is often provided by a physiotherapist (PT) through a Supported Osteoarthritis Self-Management Programme (SOASP) in primary health care (PHC) in Sweden [7]. The SOASP combines patient education with exercise and aims to support patients to cope with their disease, improve health-related quality of life (HRQoL), increase physical activity level, and reduce healthcare consumption and sick leave due to OA [7, 8]. In brief, the SOASP usually consists of two to three educational sessions aiming to provide information about OA, symptoms, coping strategies, first line and additional treatment [7]. Thereafter, patients are offered an individually adapted exercise programme to practice at home or in a PT supervised group for 6 to 8 weeks [7].

In Sweden, there is a unique opportunity to evaluate the SOASP through a national quality registry called the Swedish Osteoarthritis Registry [8]. The coverage ratio for the registry, estimated on the proportion of patients attending the SOASP that are also recorded in the registry, has been somewhere between 60 and 70% in the past few years [9]. The registry contains data on, for example physical activity level, pain and HRQoL collected through Patient Reported Outcome Measures (PROMs) [8]. However, whether the patients report to be able to cope with their disease after participating in the SOASP is not, to our knowledge, routinely evaluated to date.

Empowerment has been described as a process to gain control over decisions that affect their health and personal life [10]. Patient enablement has to do with a person’s ability to understand and cope with their disease after a consultation in health care [11, 12]. The concepts of empowerment and enablement are closely related [13–15]. Empowerment can be measured with the Swedish Rheumatic Disease Empowerment Scale (SWE-RES-23) [16] and patient enablement with the Patient Enablement Instrument (PEI) [11, 12, 17]. Both the SWE-RES-23 and the PEI can be used to evaluate patient´s ability to cope with their disease after participating in a SOASP [18].

Studies have shown an association between empowerment and HRQoL [19] in different contexts and in different diseases like cancer [20], rheumatoid arthritis [21, 22] and in OA patients after total hip or total knee arthroplasty [19]. For enablement, studies have shown an association between enablement and self-reported health [23–25] and a correlation between enablement and change in asthma-related quality of life [26]. To the best of our knowledge, there is a research gap regarding the association between empowerment, enablement and HRQoL after participating in a SOASP.

Resources in health care are limited and it is vital that they are used effectively and for those who need it the most [6, 27]. Consequently, patients suffering from chronic diseases such as OA will have to be able to take care of themselves to a greater extent in the coming years [28]. Therefore, it is crucial to be able to identify patients with the greatest need for additional support, medical care and treatment [29] and the need to predict response to treatment has been raised to tailor treatment and personalise care [30, 31]. The aim of our study was to examine change in health-related quality of life from baseline to three- and nine-months follow-up after participating in a Supported Osteoarthritis Self-Management Programme. Furthermore, to examine if empowerment and/or enablement were associated with change in health-related quality of life from baseline to nine months follow-up after a Supported Osteoarthritis Self-Management Programme.

## Methods

### Design and setting

We used data from patients with OA of the hip and/or knee participating in a SOASP in PHC to conduct this prospective observational cohort study. The study was conducted in compliance with the Declaration of Helsinki [32] and was approved by the Regional Ethical Review Board in Lund, Sweden (2015/918). The Strengthening the Reporting of Observational Studies in Epidemiology (STROBE) checklist was used as guidance when reporting this study [33] (Additional file 1). This study was registered with ClinicalTrials.gov. Identification number: NCT 02974036. Retrospectively registered 28/11/2016.

### Participants

Inclusion criteria were patients with OA of the hip and/or knee, understanding Swedish and participating in a SOASP. Patients not diagnosed with OA, i.e., having symptoms and/or pain due to other causes than OA, are not eligible for SOASP, and were thereby not invited to participate in this study. There were no other exclusion criteria. Patients were recruited consecutively between April 2016 and June 2018 and consented by the PT responsible for the SOASP at the PHC centre. All participants were given written and verbal information about the study and gave written informed consent for study participation prior to the start of the study. We included patients that had paired data, i.e., prior to the participation in the SOASP at baseline and at follow-up at nine months on HRQoL (*n* = 119), in the analysis.

### Data collection

**Fig. 1:**
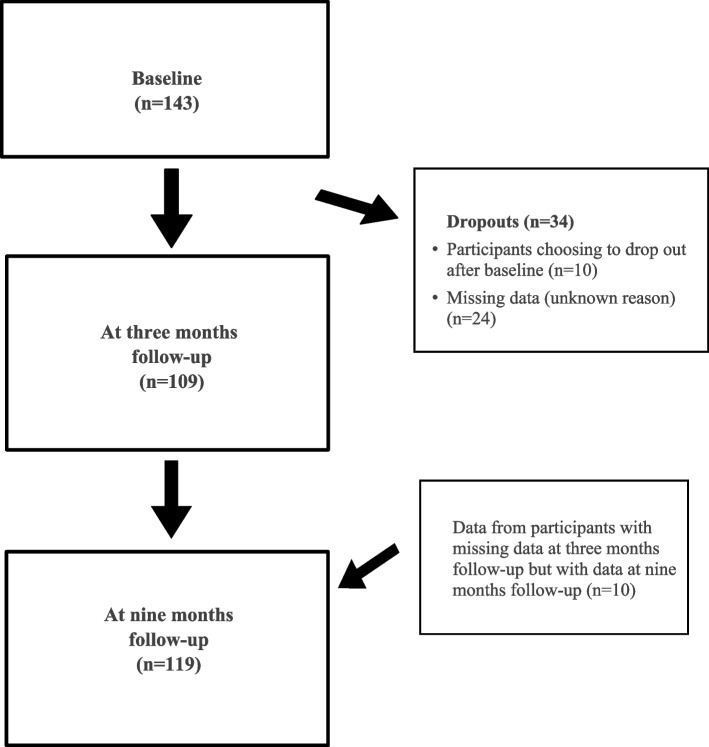
Flowchart for the selection of data for analysis in the study

Data was collected in two regions in Sweden: Region Skåne (five PHC centres, *n* = 87) and Region Blekinge (two PHC centres, *n* = 56) where the SOASP was offered in real world clinical setting. The participants answered the EuroQoL Five Dimension Five Level Scale Questionnaire (EQ-5D-5L) and the SWE-RES-23 prior to the participation in the SOASP at baseline and at follow-up at three months. The EQ-5D-5L was also answered at follow-up at nine months and the PEI was solely answered at three months follow-up. Data was collected by the PT responsible for the SOASP at the PHC centre at baseline and at three months follow-up and by the first author at nine months follow-up through a postal questionnaire. Flowchart for the data collection for analysis in the study is presented in Fig. 1 (Fig. 1).

### Outcome measures

All study data were based on PROMs. Health-related quality of life (HRQoL) was measured with the EQ-5D, which is a generic non-disease specific PROM [34–37] used frequently in OA research [38, 39]. The EQ-5D-5L consists of two parts: the descriptive part and the visual analogue scale (EQ VAS) [37]. The descriptive part consists of five dimensions: mobility, self-care, usual activities, pain/discomfort, and anxiety/depression [36, 37]. For each dimension there are five alternative answers referring to level of problems that correspond to a number (1 = no, 2 = slight, 3 = moderate, 4 = severe, and 5 = extreme/unable to) [36, 37]. Each number can be combined into a five-digit number describing 3125 unique health profiles [36, 37]. The health profiles can be represented by an index value where lower values reflect lower HRQoL and higher values reflect higher HRQoL [40]. On the EQ VAS, the patients report their self-rated health on a vertical visual analogue scale ranging from 100 (best imaginable health) to 0 (worst imaginable health) [37]. The EQ-5D-5L has been translated to Swedish [37] and has shown sufficient reliability [38] and validity [41] for use in relation to OA. The Swedish tariff was used in our study [37].

Empowerment was measured with the Swedish Rheumatic Disease Empowerment Scale (SWE-RES-23) (Additional file 2) [16]. The SWE-RES-23 consist of 23 questions with five alternative answers ranging from strongly disagree (scored 1) to strongly agree (scored 5) which is summarised as a total average score between 1 and 5 points where a higher score indicates higher empowerment [16]. The SWE-RES-23 was developed from the Swedish Diabetes Empowerment Scale [16, 42]. It was developed for rheumatic disease, and it was tested by patients with OA in the development phase [16].

Enablement was measured with the Patient Enablement Instrument (PEI) (Additional file 3) [11, 12, 17] that measures a patient’s perceived ability to understand and cope with their disease and is answered after a consultation and therefore baseline data are not possible to collect [11, 12, 17]. The PEI consists of six questions with four alternative answers: much better (scored 2), better (scored 1), same or less (scored 0), not applicable (scored 0), resulting in a possible total consultation score between 0 and 12 [11, 12, 17] where a higher score indicates higher enablement [11, 12, 17]. The basis for the development of the PEI was the idea that how the patient feels and perceives life is important when predicting outcome [17, 43]. The PEI and the SWE-RES-23 have been translated to Swedish and tested for reliability [16, 44] and for validity [16, 45]. The measurements used in this study (the EQ-5D-5L, the SWE-RES-23 and the PEI) have been described more in detail elsewhere [11, 12, 16–18, 36, 37].

### Patient partner

We involved a patient partner (PP) [46] from the Swedish Rheumatism Association to enhance the patient perspective. The PP collaborated actively on the planning of the study throughout the research process and contributed with lived experience of OA, experience of participating in SOASPs and of patient expertise in relation to the study. We used the Guidance for Reporting Involvement of Patients and the Public (GRIPP2-SF) checklist [47, 48] as a guide when reporting the PP´s involvement in our study (Additional file 4).

### Statistical analysis

Descriptive statistics are presented as frequencies and percentages for categorical data and as means and standard deviations for numerical data. Change in EQ-5D-5L from baseline to three and nine months respectively was presented with paired samples t-test. Standardised effect sizes (Cohen´s *d*, CI 95%) were analysed and categorised as small (0.2), medium (0.5) or large (0.8) [49]. First, preliminary analyses were performed to ensure there was no violation of the assumption of normality, linearity, and multicollinearity. Thereafter, a multiple linear regression was performed to assess if the SWE-RES-23 at three months follow-up and change in the SWE-RES-23 score from baseline to three months follow-up and the PEI, were associated with change in HRQoL (EQ-5D-5L index and EQ VAS respectively) from baseline to nine months follow-up after a SOASP. Dependent variables in respective model were change in EQ-5D-5L index and EQ VAS from baseline to 9 months follow-up. The total score of the SWE-RES-23 and the PEI at three months follow up and the change in in the SWE-RES-23 score during the SOASP (from baseline to three months follow-up) were entered as independent variables in model 1. To control for the potential influence of age, gender and the EQ-5D-5L score at baseline (for EQ-5D-5L index and EQ VAS respectively) these variables were entered in model 2.

A sample size calculation for a previous study using the same study sample [18] showed that to be able to detect an association corresponding to a correlation coefficient between 0.3 and 0.5 with a power of 0.80 at a chosen significance level of 0.05, 110 participants were needed. The sample size calculation for the previous study was performed with respect to multiple statistical analyses being planned on the same study sample in the present subsequent study. SAS Enterprise Guide 6.1 for Windows (SAS Institute Inc., Cary, NC, USA) was used for sample size calculation. Prior to study start, we decided to collect data from at least 140 participants to compensate for potential missing data. No imputation was made for missing values [50]. All statistical analysis was performed with IBM SPSS Statistics 27.

## Results

Baseline PROMs were answered by 143 patients. Demographic data for the study sample with paired data i.e., prior to the participation in the SOASP at baseline and at follow-up at nine months on HRQoL (*n* = 119) did not differ from the study cohort (*n* = 143) and are presented in Table 1.

**Table 1:** Sample characteristics for the total study cohort (*n* = 143) and for the study sample with paired data on health-related quality of life (*n* = 119)

	Total study cohort (*n*=143)	Study sample (*n*=119)
Gender % (*n*)		
Men	22 (32)	23 (27)
Women	78 (111)	77 (92)
Age (years)		
mean (SD)	65.9 (9.3)	66.4 (8.7)
min-max	40–90	40–90
Most affected joint % (n)		
knee	72.1 (101)	72.3 (86)
hip	25.7 (36)	25.2 (30)
hand	2.1 (3)	2.5 (3)
missing data	2.7 (3)	
BMI^a^ mean (SD)	28.9 (6.3)	28.7 (6.4)

^a^*BMI *Body Mass Index

The highest average value for the EQ-5D-5L index and the EQ VAS was observed at three months follow-up (Table 2).

**Table 2:** Descriptive data on health-related quality of life measured with the EQ-5D-5L index and the EQ VAS at baseline, 3- and 9-months follow-up and the mean change in the EQ-5D-5L index and the EQ VAS from baseline to 3- and 9-months follow-up

	Baseline (*n*=119)	3 months follow-up (*n*=109)	9 months follow-up (*n*=119)	Mean change baseline to 3 months (*n*=109)	95% CI^**c**^	***p-***value^**d**^	***d*** ^***e***^	Mean change baseline to 9 months (*n*=119)	95% CI^**c**^	***p-***value^**d**^	***d***^***e***^
Mean (SD)											
EQ-5D-5L^a^	.810 (.128)	.852 (.118)	.832 (.127)	.040	.02, .06	≤.001	.43	.022	.00, .04	.041	.19
EQ VAS^b^	68.9 (19.2)	72.7 (19.4)	71.9 (17.2)	3.5	.97, 6.1	.007	.26	3.0	-.04, 6.0	.053	.18

^a^total score range 0–1

^b^total score range 0–100

^c^CI= confidence interval for mean change baseline to 3 and 9 months respectively

^d^significance level *p*<.05, *p-*value for mean change baseline to 3 and 9 months respectively analysed with paired sample t-test

^e^ *d*= Cohen´s d (effect size)

The mean EQ-5D-5L index score increased significantly from baseline to three months corresponding to a standardised effect size (Cohen´s *d*) of *d* = 0.43, 95% CI [0.24, 0.63] (*n* = 109), and from baseline to nine months *d* = 0.19, 95% CI [0.01, 0.37] (*n* = 119). The average EQ VAS score increased significantly from baseline to three months corresponding to a standardised effect size of *d* = 0.26, 95% CI [0.07, 0.45] (*n* = 109), and from baseline to nine months *d* = 0.18, 95% CI [ 0.00, 0.36] (*n* = 119).

The self-reported average outcome for the SWE-RES-23 was 3.7 (SD 0.6) at baseline (*n* = 118) and 3.9 (SD 0.5) at three months follow-up (*n* = 105). For the PEI the average outcome was 6 (SD 3.2) at three months follow-up (*n* = 105). Neither the SWE-RES-23 nor the PEI at three months follow-up or the change in the SWE-RES-23 score from baseline to three months follow-up were associated with change in either EQ-5D-5L index (p > 0.05) or the EQ VAS (p > 0.05), and together explained 6.8% (EQ-5D-5L index) (*p* = 0.069) and 2.9% (EQ VAS) (*p* = 0.399) of the variation. When age, gender and EQ-5D baseline values were added, the models explained 34.4% (EQ-5D-5L index) (*p* = 0.000) and 42% (EQ VAS) (*p* = 0.000) of the variation, with baseline EQ-5D as the main significant predictor (Tables 3 and 4). Also, the PEI was significantly associated with change in EQ VAS (B = 1.26, 95% CI [0.25, 2.28] (Table 4, model 2).

**Table 3:** Multiple linear regression analysis for variables associated with change in health-related quality of life measured with EQ-5D-5L index from baseline to 9 months follow-up

Variables	Model 1 (*n*=103)				Model 2 (*n*=103)			
	B	95 % CI	ß	*p-value* ^*e*^	B	95 % CI	ß	*p-value* ^*e*^
PEI^a^ at 3 months	.01	-.00, .02	.18	.128	.01	-.001, .01	.16	.111
SWE-RES-23^b^ at 3 months	.00	-.05, .06	.01	.953	.05	-.001, .10	.21	.054
Change in SWE-RES-23 score^c^	.03	-.01, .08	.15	.157	.02	-.02, .06	.10	.262
EQ-5D-5L^d^ index at baseline					-.50	-.67, -.33	-.55	<.001
Age					.00	-.002, .02	-.02	.852
Gender					-.01	-.06, .03	-.05	.561
R²	.068				.344			
Adjusted R²	.040				.303			
*p-value* ^e^	.069				<.001*			

^a^PEI: Patient Enablement Instrument, measuring enablement

^b^SWE-RES-23: Swedish Rheumatic Disease Empowerment Scale, measuring empowerment

^c^Change in the SWE-RES-23 score during the SOASP (i.e., from baseline to 3 months follow-up)

^d^EQ-5D-5L: descriptive part of EQ-5D, measuring health-related quality of life

^e^significance level *α* =0.05

**Table 4:** Multiple linear regression analysis for variables associated with change in health-related quality of life measured with EQ VAS from baseline to 9 months follow-up

Variables	Model 1 (*n*=103)				Model 2 (*n*=103)			
	B	95 % CI	ß	*p-value* ^*e*^	B	95 % CI	ß	*p-value* ^*e*^
PEI^a^ at 3 months	.82	-.44, 2.07	.15	.199	1.26	.25, 2.28	.23	.015
SWE-RES-23^b^ at 3 months	-1.61	-9.60, 6.40	-.05	.690	5.08	-1.56, 11.72	.16	.132
Change in SWE-RES-23 score^c^	3.03	-3.98, 10.05	.09	.393	1.21	-4.31, 6.73	.04	.665
EQ VAS^d^ at baseline					-.55	-.70, -.41	-.64	<.001
Age					-.18	-.47, .12	-.10	.240
Gender					-.91	-7.14, 5.32	-.02	.772
R²	.029				.420			
Adjusted R²	.001				.384			
*p-value* ^*e*^	.399				<.001*			

^a^PEI: Patient Enablement Instrument, measuring enablement

^b^SWE-RES-23: Swedish Rheumatic Disease Empowerment Scale, measuring empowerment

^c^Change in the SWE-RES-23 score during the SOASP (i.e., from baseline to 3 months follow-up)

^d^EQ-VAS: visual analogue scale part of EQ-5D, measuring health-related quality of life

^e^significance level *α* =0.05

## Discussion

In our study, the patients reported relatively high HRQoL, as measured with the EQ-5D, at baseline and at follow-up at three and nine months. The change in HRQoL between baseline and three and nine months respectively corresponded to a statistically significant but small effect size regarding both the EQ-5D-5L index and the EQ VAS. To the best of our knowledge, this is the first study to examine the association between empowerment, enablement and change in HRQoL among patients participating in a SOASP. We found that empowerment and enablement were not associated with change in HRQoL in this context.

The aims of the SOASP are to empower and enable OA patients to better cope with their disease and to increase HRQoL [7, 8]. The patients in our study reported relatively high HRQoL already at baseline indicating that the patients were doing rather well at time for inclusion in our study and therefore a ceiling effect might impact further increase in HRQoL and the observed change over time may be due to regression to the mean. Even so, HRQoL increased somewhat at three months and was still higher at nine months follow-up than at baseline. This outcome pattern of self-reporting HRQoL after participating in a SOASP is in line with previous reports [51, 52] and is promising since it indicates that the aim of the SOASP to increase HRQoL is somewhat achieved. However, the effect size regarding HRQoL was small which might not be surprising given the high baseline values.

Empowerment and enablement at three months follow-up and change in empowerment from baseline to three months could were not associated with change in HRQoL from baseline to nine months follow-up in this context. The baseline values for the EQ-5D-5L were entered in the adjusted models since these might have a confounding effect. No clear confounding effect was shown, but the baseline levels turned out to be the strongest independent predictors of the change in outcome, with a negative association which can at least partly be explained by the regression to the mean phenomena. Patients self-reporting lower values on the EQ-5D-5L at baseline might have more potential to increase in HRQoL from baseline to nine months follow-up. On the other hand, patients self-reporting higher values on the EQ-5D-5L already at baseline might increase less since there is less room for improvement. Our results are in line with a recently published systematic review [53] showing that patients with hip and/or knee OA who self-reported higher pain and poorer physical function at baseline benefited more from exercise, which is a part of SOASP, than patients who self-reported lower pain and better physical function at baseline. Moreover, HRQoL vary for men and women [52, 54, 55] and in different ages [54, 56] so for that reason age and gender were entered as covariates. However, in this context, age and gender were not associated with the change in HRQoL.

Patients that self-report lower levels of HRQoL might have most to gain from participating in an empowering and enabling intervention like a SOASP. Thus, the results of our study might have been different with such an OA population i.e., those with lower levels of HRQoL. This idea was acknowledged by our PP who also highlighted that patients that choose to seek care and to participate in a SOASP might be more optimistic, able and empowered than the average patient and that a certain amount of empowerment and enablement is needed to be able to take care of oneself and actively seek health care. More research is needed when it comes to empowerment, enablement and HRQoL in relation to SOASP. In our study, the patients reported relatively high values on empowerment and enablement [11, 57] at all measuring points. It is a challenge to reach patients who might benefit most from participating in a SOASP and to include them in research. More effort should be made to reach out to OA patients and to make treatment more appealing and perhaps more tailored. A closer cooperation with patient partners and OA patient organisations might be part of a solution of reaching more OA patients and in developing OA treatment.

The SWE-RES-23 was developed in 2012 [16] and is a relatively new instrument that has not been widely used in studies yet. To our knowledge, the SWE-RES-23 has not previously been used in research about SOASP. The approach in published studies [21, 22] differs from ours so the possibility for comparisons with other studies is limited. Therefore, the current study will contribute by providing further knowledge as a basis for future studies.

The PEI is considered to be the “gold standard” when measuring enablement [58]. In our study, enablement was measured at three months follow-up. The PEI was developed to be answered after a consultation [11, 17] and patients self-report their perceived change in enablement compared to before the consultation [11, 59]. Therefore, as there is no baseline measure [59] it is thus not possible to compute a change in enablement between baseline and three months follow-up. Again, the PEI value reported in our study is moderate to high and this tends to support the argument that the participants started with relatively good understanding and coping ability, perhaps motivating them to enter the SOASP. There is also a risk of recall bias as the PEI was answered at three months since patients might not accurately recall their baseline status [59] and this has to be taken in consideration when interpreting the results. Subgroup analysis was not possible in our study, but the PEI has shown to differ by age and gender [17].

In future studies, an even further longitudinal perspective than in the present study, on all the PROMs prior to, and after the SOASP should be taken to study the association between patient empowerment, enablement and HRQoL. A larger longitudinal sample would also permit subgroup analysis regarding gender, level of education, socioeconomic status, most affected OA joint and body mass index.

## Strengths and weaknesses

A strength with the study was that data was collected in clinical practice which supports external validity. Another strength was that the response rate was high, and the amount of missing data was minimal. This might be explained by the fact that data collection was performed by PTs’ used to collecting PROMs linked to the SOASP. A PP was involved in the whole research process adding deeper meaning and relevance to the study. In the planning phase, the PP affirmed the importance of the research question, reviewed the questionnaires used in the study and commented on the proposed data collection methods. When analysing and interpreting data, the PP was consulted to see if she interpreted the results in the same way as the research team. We believe that our study became more patient-centered since we involved a PP who added valuable feedback and advice in all stages of the study process.

The observational design, and lack of control group means that no casual inferences can be made. The observed change over time in HRQoL may be due to regression to the mean.

It is also important to bear in mind when interpreting the results of our study that only Swedish speaking patients were included in the study making the sample rather homogenous and selected which is a limitation for generalizability to OA populations. In this study, data was collected following usual care and information about severity of OA pathology and/or radiographic classification of OA was neither available nor applied to the study sample collected in clinical setting. In addition, to date it is not known how long achieved level of empowerment, enablement and/or HRQoL lasts. Therefore, the timepoint for measuring the PROMs might matter for the results. Moreover, the PEI was only measured at a single timepoint, and repeated measuring points might provide an advantage over single timepoint measurement regarding prediction through the ability to capture change over time and less sensitivity to measurement error [60]. Hence, with additional repeated measures in the present study, the results of the predictive analyses might have been different. As is common in such “in vivo” clinical studies we do not have information regarding the reasons for patients not participating in the study.

## Implications

In contemporary practice patients included in a SOASP might not be representative of the average OA patient and therefore more effort should be made to reach out to struggling OA patients who may be less motivated and especially in need of support. More research is needed to identify OA patients with the greatest need for additional support and to find outcome measures to predict outcome for OA treatment.

## Conclusions

The results of our study showed increasing HRQoL for patients with OA after participating in a SOASP. Empowerment and enablement as measured with the SWE-RES-23 and the PEI respectively were not associated with change in HRQoL after a SOASP.

### Supplementary Information

**Additional file 1.** STROBE Statement—Checklist of items that should be included in reports of cohort studies

**Additional file 2.**

**Additional file 3.**

**Additional file 4.**

## Data Availability

The dataset generated and analysed during the current study are not publicly available due to the ethics approval and Swedish law (but are available from the corresponding author on reasonable request).

## Abbreviations

OA: Osteoarthritis
PT: Physiotherapist
SOASP: Supported Osteoarthritis Self-Management Programme
PHC: Primary Health Care
HRQoL: Health-Related Quality of Life
PROM: Patient Reported Outcome Measure
SWE-RES-23: Swedish Rheumatic Disease Empowerment Scale
PEI: Patient Enablement Instrument
EQ-5D-5L: EuroQoL Five Dimension Five Level Scale Questionnaire
PP: Patient Partner
