# Supplementary figures and images for: Empowerment and enablement and their associations with change in health-related quality of life after a supported osteoarthritis self-management programme – a prospective observational study
Source: Arch Physiother. 2023 Sep 22;13:18. doi: 10.1186/s40945-023-00172-7 (PMC10514979; doi:10.1186/s40945-023-00172-7)

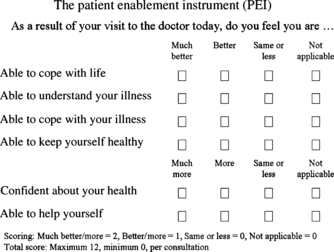

Supplement: Supplementary file 3 — Additional file 3. [file 40945_2023_172_MOESM3_ESM.docx]
