# Supplementary material for: Empowerment and enablement and their associations with change in health-related quality of life after a supported osteoarthritis self-management programme – a prospective observational study
Source: Arch Physiother. 2023 Sep 22;13:18. doi: 10.1186/s40945-023-00172-7 (PMC10514979; doi:10.1186/s40945-023-00172-7)
Supplement: Supplementary file 4 — Additional file 4. [file 40945_2023_172_MOESM4_ESM.docx]

**GRIPP2 short form**

| **Section and topic** | **Item** | **Reported on page No** |
| --- | --- | --- |
| 1: Aim | Report the aim of PPI in the study | 7 |
| 2: Methods | Provide a clear description of the methods used for PPI in the study | 7 |
| 3: Study results | Outcomes—Report the results of PPI in the study, including both positive and negative outcomes | 11-13 |
| 4: Discussion and conclusions | Outcomes—Comment on the extent to which PPI influenced the study overall. Describe positive and negative effects | 11–13 |
| 5: Reflections/critical perspective | Comment critically on the study, reflecting on the things that went well and those that did not, so others can learn from this experience | 12-13 |
